# Prerequisites for Infection Prevention Interventions During the Intraoperative Phase From the Perspective of Operating Room Nurses—An Integrative Review

**DOI:** 10.1002/nop2.70498

**Published:** 2026-03-19

**Authors:** Ida Markström, Kristofer Bjerså, Margareta Bachrack‐Lindström, Gunilla Hollman Frisman, Karin Falk‐Brynhildsen

**Affiliations:** ^1^ Department of Health, Medicine and Caring Sciences, Division of Nursing Sciences and Reproductive Health Linköping University Linköping Sweden; ^2^ Department of Anesthesiology and Intensive Care Vrinnevi Hospital Norrköping Sweden; ^3^ Department of Surgery, Clinical Sciences, Sahlgrenska Academy University of Gothenburg Gothenburg Sweden; ^4^ Faculty of Medicine and Health. School of Health Sciences Örebro University Örebro Sweden

**Keywords:** antisepsis, infection control, integrative review, nurse, operating room, patient safety, perioperative, postoperative complications, prevention, surgical site infections

## Abstract

**Aim:**

To explore the prerequisites for operating room nurses to carry out infection prevention interventions during the intraoperative phase.

**Design:**

Integrative review.

**Review Method:**

Whittemore and Knafl's method guided this integrative review. Database searches were conducted in PubMed, CINAHL, EBSCO, Embase and Web of Science's Core Collection. The initial search took place on April 12, 2022, with complementary searches on November 11, 2024.

**Results:**

Four categories emerged from the analysis of the 18 included studies. These categories encompassed the intrapersonal prerequisites of the operating room nurses, interpersonal prerequisites within the operating room team, adherence to guidelines and supportive conditions.

**Conclusions:**

Prerequisites for infection prevention interventions during the intraoperative phase included the professional competence of operating room nurses, a supportive team and sufficient resources provided by management, such as adequate equipment and time for planning and implementation. Ensuring patient safety requires operating room nurses to embrace their leadership roles and adhere to evidence‐based recommendations.

**Reporting Method:**

The PRISMA 2020 statement.

**Implications for the Profession and Patient Care:**

For patient safety, management should strive to ensure that the necessary prerequisites are in place to provide safe intraoperative infection prevention.

**Impact:**

This study offers valuable insights for the global community of operating room nurses, operating room team members and managers regarding the essential prerequisites for safe infection prevention for vulnerable patients during the intraoperative phase. No patient or public contribution was included in this study.

**Patient or Public Contribution:**

No patient or public contribution was included in this study.

## 1 Background

Patients undergoing surgery are exposed to significant risks and are particularly vulnerable to surgical site infections. Surgical site infections are hospital‐acquired infections that occur after surgery at the incision site or in deeper tissues where the surgery took place (Centers for Disease Control Prevention 2024). Surgical site infections pose a significant threat to patient safety and may cause suffering in the form of physical disability, additional surgical procedures, reduced quality of life, morbidity and mortality (Badia et al. 2017; Andersson et al. 2010; Brown et al. 2014). More than 131 million surgical procedures are performed worldwide every year (Meara et al. 2015). The global pooled incidence of surgical site infection among patients was 2.5% (Mengistu et al. 2023). The annual incidence of surgical site infections in the United States is approximately 1%, with approximately 8000 directly related deaths (Scott 2009). In 2017, the European Union countries reported rates of around 1.5% from a total of approximately 649,000 surgical procedures (European Centre for Disease Prevention and Control 2019). In developing countries, the numbers are higher, with over 30% of surgical patients developing surgical site infections each year (Allegranzi et al. 2011). Surgical site infections result in significantly increased clinical workloads and economic burdens (Badia et al. 2017). In the United States, they are the costliest of the hospital‐acquired infections, with an estimated annual cost of $3.3 billion, extending the length of the hospital stay by 9.7 days (Zimlichman et al. 2013).

Surgical site infections are primarily caused by bacteria entering the open wound during surgery and spreading via the bloodstream or originating from infections at other anatomical sites (Greene 2012). The development depends on the interaction between the bacterial load, bacterial virulence and the patient's resistance to infection (Mockford and O'Grady 2017; Owens and Stoessel 2008). There are several factors associated with causing surgical site infection, such as the patient's physical health, the type of surgery and the environment (Owens and Stoessel 2008; Gaynes 2000; Fry 2011).

By adopting a team‐based approach during the perioperative phase (which includes the time before, during and after surgery), it is possible to prevent up to 50% of these infections (Liu et al. 2018; Schreiber et al. 2018). Evidence‐based guidelines for the prevention of surgical site infections encompass a range of recommended infection control measures, including antiseptic prophylaxis, prevention of hypothermia, antimicrobial prophylaxis, glucose regulation, appropriate ventilation and skin preparation (Keenan et al. 2014; Berríos‐Torres et al. 2017; National Institute for Health and Care Excellence 2019).

The intraoperative phase, defined as the period from the patient's admission to the operating room until their transfer to the recovery unit, is considered a high‐risk period for contamination (Mockford and O'Grady 2017; Benze et al. 2021; Savage and Anderson 2013). During this phase, the surgical wound is exposed, making infection prevention particularly critical. An aseptic environment and sterile equipment are fundamental (Rothrock 2018). Infection prevention interventions are implemented during this phase with the aim of preventing contamination by environmental microorganisms or skin flora. In many countries, the operating room nurse is responsible for maintaining aseptic conditions and implementing infection prevention interventions during the intraoperative phase. This includes identifying the risk of infections, preparing the surgical site, covering the skin and ensuring a sterile field (Von Vogelsang et al. 2019). However, practices may vary internationally and the extent of this responsibility can differ between countries (Rothrock 2018; Riksföreningen för Operationssjukvård 2020; Association of Perioperative Registred Nurses 2017). Other nursing roles that may have aseptic responsibilities in the intraoperative phase include perioperative nurses, surgical nurses and scrub nurses (Benze et al. 2021).

The operating room is a high‐risk environment in which different professional groups with different specialisations, foci and training must work together in sometimes stressful situations (Hull et al. 2011). Research has shown that the OR environment and team dynamics can affect the quality of care and patient safety (Koch et al. 2020; Mentis et al. 2016; Hu et al. 2012). While guidelines for infection prevention exist, effective implementation depends on enabling factors within the clinical environment. One study showed that team dynamics, organisational conditions and environmental factors influence OR nurses' ability to carry out infection‐preventive skin preparation (Markström et al. 2020). These findings point to the importance of understanding prerequisites. A prerequisite is defined as something that is required as a prior condition for something else to happen or exist (Oxford Languages 2024). There is limited knowledge about the prerequisites necessary for operating room nurses to perform infection prevention interventions safely and effectively during the intraoperative phase. Understanding what these prerequisites are is essential for identifying barriers, supporting evidence‐based practice and enhancing patient safety. The findings may help reveal both barriers and enabling factors in surgical settings, thereby contributing to safer perioperative care and improved patient outcomes on both global and national levels. This integrative review aims to explore the prerequisites for operating room nurses to carry out infection prevention interventions during the intraoperative phase.

## 2 Methods

An integrative review was chosen for its ability to synthesise diverse study designs and provide comprehensive insights into the multifaceted prerequisites for infection prevention in perioperative nursing. Whittemore and Knafl's review method guided this integrative review, which was conducted in five stages: problem identification, a literature search, data evaluation, data analysis and presentation (Whittemore and Knafl 2005). The study protocol was retrospectively registered on the Open Science Framework (OSF; https://osf.io/nsmkz/).

### 2.1 Problem Identification

There is limited knowledge about the prerequisites necessary for operating room nurses to perform infection prevention interventions during the intraoperative phase. The review's aim and question were guided by the Population, Concept and Context framework (Peters et al. 2020) as follows:

- P (Population): Operating room nurses and other nursing professionals whose roles involve aseptic responsibilities (e.g., scrub nurses, perioperative nurses and surgical nurses)
- C (Concept): Experiences of prerequisites for infection prevention interventions
- C (Context): Infection prevention interventions during the intraoperative phase

Based on this, the research question was formulated as follows: ‘What are the prerequisites for operating room nurses to carry out infection prevention interventions during the intraoperative phase?’

### 2.2 Inclusion and Exclusion Criteria

English‐language, peer‐reviewed scientific studies were included if they addressed prerequisites for infection prevention interventions during the intraoperative phase from the perspective of operating room nurses. In this review, an operating room nurse refers to a registered nurse with aseptic responsibility during surgery, including operating room, scrub, perioperative and surgical nurses. Infection prevention is understood as intraoperative measures aimed at preventing surgical site infections. Prerequisites are defined as the conditions or requirements that must be fulfilled to enable operating room nurses to perform infection prevention interventions during the intraoperative phase. Studies were excluded if they were reviews, guidelines, theses, abstracts, letters, non‐peer‐reviewed articles, or if findings specific to the described nursing roles could not be extracted.

### 2.3 Literature Search

**FIGURE 1:**
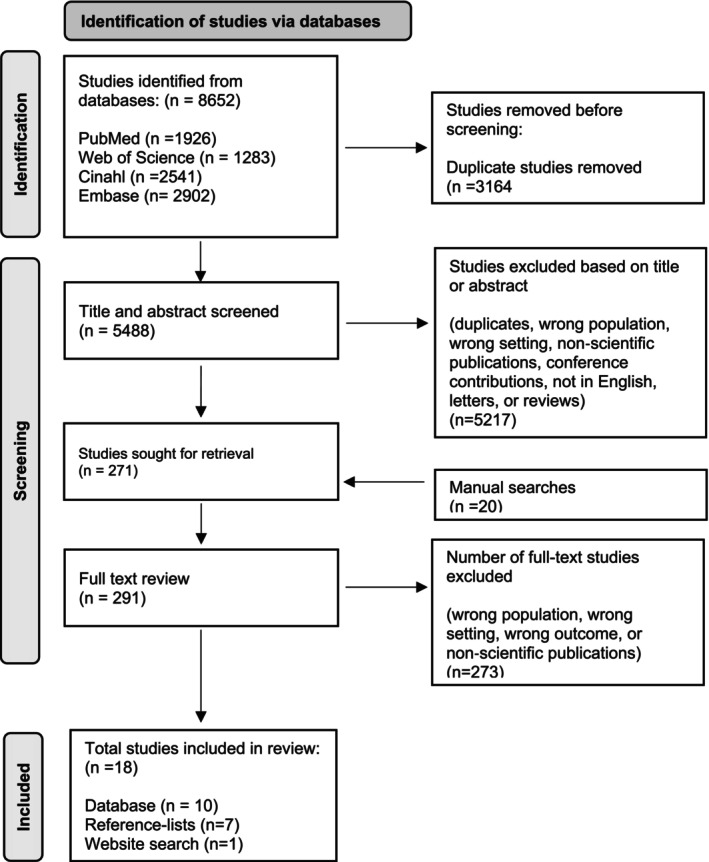
Preferred reporting items for systematic reviews and meta‐analyses (PRISMA) flow diagram.

Based on the research question, a systematic search strategy was organised using searches of academic databases, manual screening of reference lists (backward chaining) and citation searches via Google Scholar (forward chaining), along with targeted internet searches. Computer‐assisted searches with both thesaurus terms and keywords in four academic databases were performed. The search terms used were terms related to patient safety and operating room nursing (specific search strategies are outlined in Table S1). The search started in PubMed, followed by searches in the Cumulative Index of Nursing and Allied Health Literature (CINAHL, EBSCO) and Embase. Free‐text keyword searches were employed in Web of Science's Core Collection. Only studies published in English were included, and there were no restrictions based on publication date or methodology. The initial database searches were conducted on April 12, 2022, with complementary searches on November 11, 2024. A total of 8652 studies were identified through the database searches and exported to EndNote (version X9 and 21). After removing 3164 duplicates, 5488 studies remained for screening. A flow diagram of the screening process is presented in Figure 1 (Page et al. 2021). The studies were divided among the authors and screened by title and abstract. For validation, the first author reviewed the titles of all the excluded studies, and a few were brought up for a second review and discussion in the research group. After the title and abstract screening, the studies were divided among the authors and reviewed in their entirety based on their relevance to the research question. Subsequently, the authors exchanged articles with each other. Following discussions, consensus was reached, and it was ultimately agreed to include 10 studies from the database searches in the analysis.

Additional manual screening of reference lists (backward chaining) and citation searches via Google Scholar (forward chaining) of the ten articles was performed. Furthermore, from April 2022 to November 2022, the first author received weekly updates from PubMed for new research in the search area. During the same period, the first author also conducted weekly targeted web searches through www.google.com using the combination of the words ‘operating room’ (or synonyms), ‘nurse’ and ‘patient safety’. After further discussion in the research group, the additional searches generated eight studies. This resulted in a final inclusion of 18 studies in the integrative review (see Figure 1).

### 2.4 Data Evaluation

Quality appraisals of the included studies were performed using the *Critical Appraisal Skills programme for qualitative studies* and the *Critical Appraisal Skills programme for Descriptive/Cross‐Sectional Studies* (Critical Appraisal Skills Programme 2024a, 2024b). The appraisal forms consisted of 10 and 11 questions, respectively. Each question could be answered by one of three alternatives: Yes, Can't tell/Not applicable, or No. All five authors performed the assessments independently. Due to co‐authorship, one of the authors did not evaluate Wistrand et al. (2018) and Wistrand et al. (2022). After completing individual assessments, the authors came together to discuss their findings and integrate their evaluations into a unified consensus (the combined assessment is presented in Table S2). The appraisals classified nine studies as high‐quality qualitative research (Björn and Lindberg Boström 2008; Lingard et al. 2004; Nordström and Wihlborg 2019; Nyberg et al. 2022; Sandelin and Gustafsson 2015; Sandelin et al. 2019; Alfredsdottir and Bjornsdottir 2008; Silén‐Lipponen et al. 2005; Duff et al. 2022) and two as high‐quality quantitative studies (Wistrand et al. 2018, 2022). Two qualitative studies were assessed as low quality, primarily due to insufficient methodological description (Timmons and Tanner 2005; Bastami et al. 2022). Bastami et al. (2022) were critiqued for weaknesses in the application of their analytical approach, whereas Timmons and Tanner (2005) provided no description of their analytical process. Furthermore, Timmons and Tanner (2005) demonstrated limited transparency regarding the relationship between interviewer and informant and failed to articulate a clearly defined research aim. Subsequently, the authors engaged in deliberations to reach consensus on the significance, methodological quality and potential sources of bias within each study, which informed the decisions regarding their inclusion in the review. Although certain limitations were identified, all studies were retained as their findings contributed valuable insights into the prerequisites for infection prevention that were pertinent to the research question.

### 2.5 Data Analysis

Raw data, including authors, year, country, study design, study aim, sample population, data collection methods, analysis approach and relevant meaning units from the studies' findings section, were extracted and organised into a data collection form by the first author (see Table 1). All five authors read, discussed and approved the extraction of meaning units. A constant comparative method, as described by Whittemore and Knafl (2005), was used. Three of the authors (an operating room nurse, a registered nurse, and a critical care nurse), all with experience with qualitative data and two with previous experience with the analysis method, performed the coding together. All were aware of the risk of personal judgement when researching one's own field of practice, and this risk was repeatedly and critically discussed (Patton 2002). The first meaning unit underwent analysis and was labelled with a code; subsequent meaning units were then compared to the first, receiving a new code if their content did not align. This process continued until all meaning units were coded. The entire research team discussed, reviewed and compared the codes to identify patterns, similarities and connections. This collaborative effort resulted in the emergence of four categories.

**TABLE 1:** Characteristics and summary of included studies.

Author (year), country	Purpose	Design	Methods; sample, setting, data collection, analysis method	Extracted findings from included studies
Aholaakko (2011), Finland	To explore aseptic practise‐related stress among surgery nurses	Explorative interview study	Sample: 31 intraoperative surgery nurses* * = The nurses varied their roles so that in every other operation they worked as a ‘scrub nurse’ Setting: One surgery department at Helsinki University Central Hospital in Finland Data collection: Stimulated‐recall interviews, 31 operations were videotaped and used as stimuli Analysis method: A membership categorization device analysis by Baker	*The SNs had deep personal feelings and adjusted their AP according to the person they were working with*: *I personally have a more critical attitude with surgeons*΄ *AP*. *And besides this I have to agree, that those who you know to have … a kind of… aseptic looseness… you pay more attention to… but with those you know and who have worked long… and you know are responsible… you expect that they work properly…* *After working a day with (a certain surgeon) you are exhausted because you have to be extra sharp all the time… and despite it… even though you are sharp… you recognise those situations and comment on them… and it does not help at all… So it is depressing*. ‘*I think that in principal*… *the surgeon has the final word*.’ *She also had fear of being excluded from the surgical team due to active AP feedback on the surgeon. Additionally, a beginner as a surgery nurse was*: ‘*… afraid of making the surgeon angry*.’ *The experiences of being in the focus of power‐related decision making concerning AP was not always related to the experience of the interviewee, a senior SN had experiences of*: ‘*Surgical field being a battle field*’ …as novis surgical nurses was afraid of not knowing what to do, they want to perform as good as more experienced colleagues, stressed when they need to do instruments counts and have environmental control at the same time senior SN identified stress related to the experience of her coworker or herself: *I recognise a kind of competency in behaviour of younger colleagues. Say…working experience of two to four years… a kind of feeling of competence is present… and then it bursts like a bubble when you understand things… and when you understand you respect more and are more afraid… competence is not expertise* Time stress (need to focus on the next surgery) Need to control persons with “aseptic looseness”. Persons with limitations on taking feedback or following recommendations for aseptic practise Patient‐related stress in aseptic practise was visible during operations, for example as a need to document the obesity of the patient as a potential risk for infection A young nurse was worried about harming an old patient's thin skin or had problems with patients' anatomical variances The stress was felt as positive when a surgery nurse was an Aseptic Practise specialist being in a “dream surgery team” Person‐related stress was present when a hot‐tempered surgeon; fussy co‐worker; either nurse or surgeon with limitations on taking feedback or following recommendations for aseptic practise participated in the team A nurse has to be independent and, sometimes, quite headstrong to have the right to work properly
Alfredsdottir and Bjornsdottir (2008), Iceland	To identify what threatens and enhances patient safety and how operating room nurses see their role in ensuring safety	Explorative interview study	Sample: 14 OR nurses* * = 8 OR nurses in individual interviews and 2 focus group. (Two OR nurses participated in both individual and focus groups) Setting: One university hospital in Iceland. Data collection: Individual semi‐structured interviews (*n* = 8) and focus group (*n* = 2). (A study in two stages, stage two is included in this integrative review) Analysis method: Interpretive content analysis	All participants described how prevention is always at the core of their work They also described how they had to know the background of the patient and their vulnerability and fragility that might increase risk during the operation …rely on information from the patients' records, particularly from the anaesthesia team A number of participants said that in some situations they do not have all the information required preoperatively, especially in cases of specific patient needs …better preoperative information would ensure patient‐centred nursing, continuity of care and better and more efficient preparation for the surgical operation Participants described how imbalance in staffing, which may be either under‐ or over‐staffing, may lead to unsystematic preparation or distraction The work processes are timed and, while the surgical procedure cannot be rushed, the nurses sense pressure to reduce time for preparation and time between operations
Bastami et al. (2022), Iran	To explain the lived experiences of operating room nurses' experiences with patient cares for laparotomy surgeries	Phenomenological study	Sample: 10 OR nurses Setting: A public educational hospital in Iran. Data collection: semi‐structured interviews Analysis method: Analytical phenomenological method	Aseptic technique observance in the operating room is a fundamental factor in providing indirect care for patients under surgical operation According to the participants' experiences, inadequate sterilisation of the skin and drape of the surgical site was one of the leading causes of hospital infections after the surgery *One of our colleagues preps the surgical site within less than 1 min, fills the gallipot with 7.5% betadine, but never uses it. I have noted several times, but he never pays attention and does not care about the patient's life*
Björn and Lindberg Boström (2008), Sweden	To describe the theatre nurses' work from their own perspective.	Descriptive study with Phenomenographic approach	Sample: 15 OR nurses Setting: Two hospitals in Sweden. Data collection: Interviews with open‐ended questions Analysis method: Phenomenographic technique	Theatre nurses achieve control of the situation by advanced planning and being ‘one step ahead’ ‘Control of the situation’ referred to having appropriate equipment prepared in advance of the operation, and hygiene factors such as maintaining the sterile field, while managing patient, instrument and department logistics *I foresee and plan the operation in my mind, how the patient is lying on the operating table, how to skin prep, this makes me feel at ease when we are about to start the operation* *The most important thing for a nurse is to be prepared for the operation* The lack of respect for their practice that they sometimes experienced from colleagues in other disciplines and which had a negative impact on teamwork
Duff et al. (2022), Australia	To generate insight into the phenomenon of Surgical Conscience by interpreting the experiences and perceptions of perioperative nurses.	Interpretive descriptive study	Sample: 15 perioperative nurses Setting: Perioperative nurses with a minimum of 2 years of experience in Australia. Data collection: Semi structured in‐depth interviews Analysis method: An interative process	There was a particular emphasis on the need for perioperative nurses to develop an awareness of your body in relation to the sterile field and other people and objects in the environment. (7) It was stressed that perioperative nurses needed to develop an awareness of the consequences of their actions or inactions. People need to know you actually cost the hospital money if you don't change your gloves, or you make do with something because the infection that could result is going to cost a lot more than a new gown. The actual physical and emotional toll on that patient is going to be huge. (11) *if you're the scrub you've got control. So if something's dirty and they want to keep pressing on you can say, ‘no way’. They can't physically stop you from picking everything up and then tossing it in the dirty bin and just starting over again*. (1) *The environment has a massive impact on how well you can do your job. Sometimes layout or traffic flow means you can't provide the best practice. You can say ‘don't walk through the orthopaedic theatre’, but if it's the quickest way, then that's the way they'll go*. (14) *There was frequent mention of not being backed up by a manager and the effect on a team's willingness to advocate for perioperative safety and surgical asepsis. If you or a colleague disagree with a doctor about safety and the manager comes in and sides with them, of course, next time you're going to think twice before you say anything, because you think that no‐one's going to back you up*. (4) *I think that one of the barriers [to Surgical Conscience] may well be knowledge. I would hope that all of us who work in this area are knowledgeable about maintaining a sterile field and patient safety, but I fear they're not*. (10) *I think we need strategies to be able to communicate respectfully with each other because you don't want to be confrontational. You don't need to be confrontational, and you certainly don't want to be embarrassing them, because I think that's when you start get‐ ting lots of problems*. (9) *If a person starts in a unit with relaxed sterility, then they will think that's normal. If you start in a unit where it's strict, that's how you will be*. (1)
Holmes et al. (2020), Norway	To explore Norwegian operating room nurses' perceptions of how team skills in the inter‐professional operating room team influence perioperative nursing in relation to patient safety.	Descriptive interview study	Sample: 10 OR nurses Setting: Operating departments at three general hospitals and one university hospital in Norway. Data collection: Semi‐structed interviews Analysis method: Inductive content analysis	…the OR nurses' perception that the performance of perioperative nursing is better when there are good team skills …OR nurses strive to do their tasks in a good way, even though this might be time‐consuming and tiring: “My standard is the same, independent of who the patient is, or who I'm working together with. But it's easier to achieve this if communication is good” The OR nurses perceived constructive criticism, willingness to learn, planning and a good tone as resulting in better performance of perioperative nursing The participants perceived that poor communication or situation monitoring, along with experiencing a lack of mutual support or leadership, can lead to intraoperative events such as faulty positioning or draping, lacking equipment, or forgetting a catheter or warming blanket Unnecessary communication, noise, loss of concentration, stress, insecurity and irritation have a negative influence on the performance of perioperative nursing Good communication makes it easier for OR nurses to speak up about risks to patient safety. They perceived that adverse event such as hypothermia, injury due to positioning, infection and extensive blood loss might occur partly because of poor team skills Poor communication, for example lack of information, inappropriate or unnecessary remarks, poor leadership and situation monitoring, or having to take on others' tasks, can result in delays because they lose focus on their own task *You lose some focus when you have to watch what is going on around you at the same time* *in big operations it is good when the surgeon comes in early to see everything is okay. To help find equipment, hold up the leg for skin disinfection … and at least offer to help* Poor team skills such as misunderstandings, interruptions and not being able to trust others to do their job properly can create stress, which again can increase the risk of making mistakes Poor team skills such as poor communication, including unclear messages or being yelled at, were perceived to create insecurity and a feeling of being inadequate.
Kaldheim and Slettebø (2016), Norway	To acquire knowledge about what theatre nurses, perceive as important factors in collaboration with other team members to see what factors are needed to strengthen interdisciplinary cooperation.	Explorative interview study	Sample: 8 OR nurses Setting: Four Norwegian operational units Data collection: Semi–structured interviews Analysis method: Constant comparative analysis	The theatre nurses want to be accepted as having skills and duties that are equal to the other members in the team They want to be seen and heard by the others in the team as people with tasks that are meaningful They often experience working situations where there is not always so much tolerance for having to wait for each other, and that their task must be done “quickly and invisibly” The participants want others to understand the importance of their job and to recognise that it also requires time Poor collaboration occurs when an individual only sees their own tasks and not those of others The participants say that they find it easier to work with people they know *It may be the way in which things are said, or someone raising their voice and shouting. It affects the concentration and focus of the participants in the situation, and this affects the quality of the performance of theatre nursing* *Yes, there are some situations like that where, for me at least, there will be poorer cooperation when someone gets scolded. For then you will be a little preoccupied in your head that there is a bad atmosphere here. In addition, I lose a little of my concentration* The leader should have insight into the team members' tasks and communicate the need for resources upward in the organisation
Lingard et al. (2004), Canada	To determine to what extent documented tension patterns are transferable to other institutional contexts.	Explorative validation study	Data were collected in two phases Sample phase 1: 22 OR nurses, (5 anaesthesiologists, 10 trainees, 6 surgeons) Sample phase 2: 10 surgeons (and fluctuating team members) Setting: Two small academic hospitals in Canada. Data collection: Phase 1: 8 focus group, 8 individual interviews Phase 2: Field observations Analysis method: Modified grounded theory approach	…issues of aseptic technique and patient safety influenced team communication. Nurses were almost always participants in these communication exchanges, perhaps reflecting their professional responsibility for aseptic technique. For instance: *Senior resident enters OR not scrubbed, stands inches from the sterile field. Circulating nurse turns towards resident, watches intently, frowns disapprovingly*. With only 6 of 28 observed instances (21%) involving higher tension levels, the theme of safety and sterility was not a prominent catalyst for tension
Nordström and Wihlborg (2019), Sweden	To describe the work experiences of nurse anaesthetists and OR nurses in the OR.	Phenomenographic interview study	Sample: 6 OR nurses (6 anaesthetic nurses) Setting: One university hospital and one regional hospital in Sweden. Data collection: Interviews Analysis method: Phenomenographic analysis	…they had a feeling of togetherness with other perioperative team members rather than feeling divided. An important aspect was to be acknowledged as integral members of the OR team and the recognition that every profession in the OR is a valuable part of this team Another aspect of responsibility is that all professionals must be given sufficient time to conduct their specific individual tasks They expressed a desire to be well prepared and have enough time to perform their work to the best of their ability Students and new colleagues should be given extra time to perform their tasks. When a new colleague attends, you have to tell everybody to slow down a bit so he or she has a chance to perform their part it is important to be open to changes, new procedures, new evidence, and their effects, even if it challenges the OR staff members.
Nyberg et al. (2022), Sweden	To explore aspects of patient safety practice during joint replacement surgery through assessment of operating room nurses experiences.	Explorative interview study	Sample: 21 OR nurses Setting: Three hospitals in Sweden; one university, one public general and one private orthopaedic hospital. Data collection: Semi structured interviews Analysis method: Qualitative content analysis	…stated their need for a reliable preoperative plan to ensure a safe procedure. By planning and preparing well for the procedure, they attempted to reduce the time for surgical procedure Before preparing for the procedure, they often needed to confirm the information from a computerised surgical planning system with the orthopaedic surgeon, due to occurrence of failure in updating the plan. This need to confirm the plan was perceived as unsatisfying and experienced as time‐ consuming For some participants, the main source of patient related information was derived from the surgical planning system and the anaesthesia preoperative assessment They did not find time to get information from the main health records and were thereby not routinely accessing information from the main health records …they experienced important patient information in different computerised systems in parallel a risk. There was no systematic feedback on results or complications. For example, some participants emphasised that they wanted to know the infection rates for their specific department OR management sent information about new routines and incidents by email, and these were sometimes perceived to not reach the appropriate OR personnel Collaboration, established safety controls and compliance with aseptic principles were stated as important aspects for safety practice within the team Compliance with aseptic principles was considered to vary among different professions within the team. The ORNs were expecting all team members to perform responsibly, and when this was not the case, it became a strain in the workplace for them In situations where two ORNs collaborated during surgery, they had opportunities to support and learn from each other, and thereby improve their work They kept guarding sterility throughout the entire surgical procedure by keeping an eye on the activities of other team members, which sometimes could be challenging Participants noted that the prerequisites for work in an aseptic environment were present National guidelines for preventing PJIs were established, and there was most often compliance with these One example given was a guideline to control the traffic and avoid disturbance of the ventilation by opening the doors and trying to minimise the number of persons in the OR Participants experienced that compliance with guidelines varied within the team. For example, some orthopaedic surgeons followed the guidelines more strictly than others. With an interesting surgical case, minimising the amount of personnel in the OR was disregarded by some surgeons The ORNs also emphasised a need to improve staff behaviour regarding adherence to the aseptic principles When they insisted on observing aseptic protocols, it sometimes was considered a disturbance of the flow, affecting both the surgical procedure and the whole day operation schedule When notifying others on breaks of aseptic principles, some participants perceived that they were seen as annoying *we are herd animals, we do as our colleagues do and it is uncomfortable if someone gives a reprimand or is a hygiene‐witch…Even if people might think you're irritating, I think you still get some kind of respect in that you have competence and can see that this is important. Even if you are considered awkward, you are trusted as the person who also is competent and good, good for the group and for the patient* The ORNs felt a personal responsibility to the patients and felt guilty when failing to protect the patient from harm. They protected patients by using their professional knowledge and skills and by having the confidence to speak up if the patient was put at risk in the process. Although the ORNs were well aware that many factors could have led to an infection, they felt accountable for it if a patient acquired a surgical site infection
Prati and Pietrantoni (2014), Italy	To assess attitudes about teamwork and safety among Italian surgeons and operating room nurses.	Descriptive cross‐sectional (pilot) study	Sample: 48 OR nurses (55 surgeons) Setting: One hospital in Italy Data collection: Questionnaires Analysis method Statistical analysis	The majority of the OR nurses agreed to the statement: It bothers me when others do not respect my professional capabilities (94% agreed) More than half of the OR nurses did not agree to the statement that team members frequently disregard rules or guidelines (e.g., hand washing, treatment protocols/clinical pathways, sterile field) developed for our Operating Theatre (69% disagreed)
Qvistgaard et al. (2019), Sweden	How OR nurses experience intraoperative prevention of SSIs.	Phenomenological study	Sample: 15 OR nurses Setting: Seven hospitals in Sweden Data collection: Interviews Analysis method: Phenomenological analysis	…further competencies such as experience and courage are needed to ensure prevention Prevention of SSIs depends on an open and honest atmosphere within the team, a team that allows different professionals to contribute with their unique competencies …the team members should adhere to the guidelines regarding OR hygiene “Everybody has their own responsibility inside the OR, but my job is to tell you when you are too close, when you have to change gloves, or when you need to adjust your surgical gown. You are trying to have an overview of everything that happens inside the OR and simultaneously keep the focus on what is going on during the surgical procedure” The absence of structured feedback makes it difficult when the profession seeks arguments for strengthening routines related to preventing SSIs. Therefore, measures intended to combat this invisible threat are difficult to evaluate and analyse. This lack of evidence for the effectiveness of routines results in insecurity and doubt connected to SSIs prevention measures Sterility is the alpha and omega to me; here is where my occupational pride is at stake and I cannot look the other way Awareness of risks related to SSIs is carried out by individuals who have confidence in each other and dare to confront human shortcomings Confident relations and resolute communication within the team generate favourable conditions for preventing SSIs. The invisible threat of microorganisms can be made visible to others if OR nurses use their profound knowledge to explain the connection between bacterial load and the risk of SSIs ….a lack of trust in one's colleagues creates anxiety among the team, a milieu that will not benefit the patient: “You really need your team and it's important that everybody understands why we do certain things, not just doing it because I say so” Friction among team members is evident when one or several team members are unwilling to understand other professionals' responsibilities and competencies Some people are more or less frightened of some surgeons and then you become nervous and that leads to insecurity and mistakes. For example, if a surgeon is intimidating, I might make mistakes, get nervous and take the surgical towel that I had for cleaning instruments and put it in an open wound during hip replacement Confronting colleagues irrespective of their position requires a security in one's own competence and a security in the team's willingness to hear potentially uncomfortable feedback, competencies that develop with experience Effective leadership helps team members develop confidence in organisational structures and offers stability for the team members. Managing both team collaboration and organisation are intertwined and clearly related to intraoperative prevention of SSIs. OR nurses often feel their contributions are minimised. The balance between the legitimacy of OR nurses and the authority of the traditional hierarchy, which places surgeons at the top, is fragile Prevention of SSIs often end up being a secondary priority, a lack of commitment that often leaves OR nurses feeling ignored “You can get so tired of yourself and you feel like a disc that repeats itself over and over again, but you can't give up and capitulate to what you believe is correct. Who will take an interest in SSI prevention if not me, no one would care about that” OR nurses reside in their responsibility to ensure that team members follow hygienic guidelines inside the OR
Sandelin and Gustafsson (2015), Sweden	To describe operating theatre nurses' experiences of teamwork within the surgical team in regard to achieving patient safety.	Descriptive interview study	Sample: 16 OR nurses Setting: Four hospitals in Sweden; two urban and two hospitals in rural regions. Data collection: Interviews Analysis method: Content analysis	A brief meeting (with the patient before surgery) facilitated OTNs to be better prepared for safe nursing care Interdependent collaboration with surgeons was reached when OTNs experienced respect as equal co‐workers and were involved and engaged as key partners, and spoken to with respect for their professional skills OTNs were totally dependent on nurse assistant (NAs) willingness to collaborate. The collaboration of the OTNs and NA was characterised by leadership and the NA was perceived as the OTNs' ‘right hand’ OTNs believed that friendly leadership was necessary in the collaboration as this would generate a willingness to follow OTNs' instructions efficiently Sometimes surgeons were unable to control their tantrums with consequences of other team‐members' knowledge and skills tended to decrease because of the strained atmosphere In order to be able to trust unfamiliar or inexperienced NAs, OTNs interrogated them about nursing knowledge and skills. This was necessary for the planning and the performance of nursing care and involved guiding in a polite and friendly way to do the right thing at the right
Sandelin et al. (2019), Sweden	To describe operating theatre nurses' experience of preconditions for safe intraoperative nursing care and teamwork.	Descriptive interview study	Sample: 16 OR‐nurses Setting: Four hospitals in Sweden; two urban and two hospitals in rural regions. Data collection: Interviews (reanalysed data) Data analysis: Content analysis	…operating theatre nurses (OTN) met patients preoperatively for a conversation about their health status and needs, as well as details about the surgical intervention. In these cases, OTNs expressed that they were well prepared with adequate information from the primary source for decision‐making of care activities for safe intraoperative nursing care OTNs' described frequent experiences of obtaining only brief, incomplete and fragmented information about the patients' health situations and their upcoming surgical interventions The documentation in the computerised systems was not complete, due to restrictions from codes and measures in the systems when not informed, they had to phone the surgeon in order to be properly prepared for the intervention they preferably wanted to be prepared for each patient's operation the day before For the most part, because they were moved around between different surgeries in their daily work, OTNs would not read the patient's record until the patient was confirmed and transported to the OT department. This meant that they did not have a chance to be completely prepared for each patient's surgery *Also, even when an operation was delayed because of an anaesthesia procedure, OTNs felt they were the ones to blame. Sometimes it takes time for the anaesthesia personnel, when the patient has a complex health situation, and I feel stressed doing the skin disinfection and the sterile draping …and the surgeons enter and wonder what you are doing and why it has taken such a long time* OTNs explained they needed to have high standards of personal professional skills and knowledge to be able to offer patients safe perioperative nursing care They also depicted their responsibility for the hygiene and aseptic care environment as well as, security controls of the sterile surgical equipment and the patient's well‐being and safety. OTNs' best experience of safe and efficient work occurred in situations where two colleague OTNs collaborated during surgery OTNs described the importance for ensuring patient safety of nurse leadership holding clear standards, routines and operational goals. When first‐line managers were invisible and uncommitted, the OT department was described as lacking standards and routines
Silén‐Lipponen et al. (2005), Finland, United Kingdom and Unites states of America	OR nurses' experiences about potential errors and error prevention in operating room	Interview study	Sample: 30 OR nurses Finnish (*n* = 10), American (*n* = 10) British (*n* = 10) Setting: OR departments in Finland, UK and USA (part of larger international research project) Data collection: Interviews Analysis method: content analysis	The need to manage multiple, simultaneous demands while providing high‐quality care imposed a continuous pressure on nurses Arguments during operations could lead to overheated feelings and, thus, jeopardise patients' safety by causing errors. Therefore, nurses forced themselves to remain undisturbed and to keep up sustained working Confidence about the individual team members' skills made advanced preparation possible Teams familiar with their members could pool their strengths, anticipate each other's needs (even from gestures), exchange roles across professional boundaries and, thus, minimise the occurrence of errors Contact with careless or risky behaviour: “*The instrument was gas‐sterilised but had not been aerated. I told him [the surgeon] that you cannot use it because of what it causes to human tissues is the same as a microwave oven. Still, the surgeon insisted on having it. Then the situation was taken out of my hands, and I had to write a case report about serious misbehaviour that compromised the safety of a human being*” *We had a very hard time after that incident for a long time. The team leaders still made me scrub and I didn't open my mouth during the operation. I only said ‘Yes sir’, but none of the kind of friendly conversation that normally goes on when you know each other*. *Faultlessness, which often comes with experience, is not something you can teach somebody. It grows with you, as you develop a trusting relationship with your colleagues. A great deal about teamwork is knowing people as individuals and knowing how to get the best out of them*.
Timmons and Tanner (2005), United Kingdom	To explore the emotional labour in an operating theatre nurses context	Ethnographic study	Sample: 12 OR nurses (8 other professions) Setting: Five hospitals in UK Data collection: Observations *n* = 20 and individual interviews *n* = 20 Analysis method: Not described	…it was their responsibility to ‘look after’ the surgeons rather like an air hostess or a party hostess Not upsetting surgeons describes actions which nurses refrained from undertaking to prevent antagonising surgeons …nurses might have tolerated poor practice rather than antagonise surgeons even though the poor practice was to the detriment of unconscious patients (Timmons and Tanner 2005) *The surgeon walks into the theatre. He is carrying his coffee mug and eating a roll. This contravenes theatre infection control policies. None of the nurses say anything to him* *Surgeon comes into theatre, he is not wearing a hat. This contravenes the theatre dress code and presents a risk of infection. The nurses, including Nurse W, don't say anything. Another surgeon comes in, looks at the surgeon with no hat and says ‘What, no hat, is this a new rule?* In the following example, the interviewer asked a nurse why she poured ether over some swabs for a surgeon? (Ether, a hazardous substance, is banned from theatre departments): *He likes using [ether]. “We have to get pharmacy to supply it especially for him. Yes, I know we shouldn't be using it”*. The nurses would accommodate surgeons' demands even if they did not agree with them: *The instruments had been set up and we had to wait about 30 min for the patient. When the patient came in and they were about to start operating, the surgeon asked the Sister if these were the same instruments? She said ‘Yes, but they are all right’. The surgeon said ‘I want fresh instruments’. The nurse got new instrument trays out. Later, the nurse said to me ‘I didn't need to change them but I wasn't going to argue with him’*
Wistrand et al. (2018), Sweden	To describe the daily interventions Swedish operating room nurses perform to prevent SSIs following national guidelines	Descriptive cross‐sectional study	Sample: 890 OR‐ nurses Setting: OR‐nurses from 64 hospitals in Sweden. Data collection: Web‐based questionnaire Analysis method: Descriptive statistics	Most of the nurses responded that they had learned to perform patient skin disinfection from their supervisors (another OR nurse) or at the clinical practice during in‐service education (48.9%; *n* = 435), while 41.7% *n* = 371 learned the technique from the educator at a university. The remaining 9.4% of the nurses stated either that they had learned it from colleagues or the Handbook for Healthcare Workers or that they did not remember Almost all of the nurses, 96.3% (857/890), stated that guidelines existed at their OR department for how to perform a pre‐operative hand disinfection. The majority of the nurses reported that they performed the pre‐operative disinfection of the patient for two to 5 min. Most, 41.1% (*n* = 366), often let the skin dry before draping; and to enhance adherence of the drapes to the patient's skin, 34% (*n* = 303) of the nurses often wiped the skin dry in the site where the drapes should adhere using sterile paper towels 40.4% (*n* = 360) did not know if they had any guidelines regarding the use of double gloves, and 37.1% (*n* = 330) stated that they knew they did not have any.
Wistrand et al. (2022), Sweden	To explore interventions that Swedish operating room nurses considered important for the prevention of bacterial contamination and surgical site infections.	Descriptive cross‐sectional study	Sample: 890 OR‐ nurses Data collection: Web‐based questionnaire with an open‐ended question (from part II, analysis of the open‐ended question) Analysis method: Summative content analysis and descriptive statistics	Aseptic technique was maintained during surgery by keeping the sterile goods sterile, removing the draping after the dressing was applied, supervising other persons in the surgical team to ensure that they did not contaminate anything in the sterile field, and quickly replacing any contaminated item with a new, sterile one important for the hygiene level in the OR to be satisfactory and the doors of the OR to be kept closed; or, at least, opened only when absolutely necessary during preparation for surgery and the surgery itself *Use the phone in the OR as your means of communication [with staff outside of the OR], do not run in and out. Plan your work and make sure that the equipment you might need is in the OR, use reach‐through cabinets as much as possible* The nurses described a calm environment, few people and no opening of doors as important factors in order to minimise bacterial air contamination The nurses believed that it was important for all personnel working in the OR to be dressed appropriately in tightly woven clothes or clean air suits, including using a mask and helmet, with sterile gowns and gloves for the personnel actively working with or around the surgical area… The nurses stated that they felt it best to set up and cover the sterile goods before the patient arrived at the OR if possible, and that it was important for the preparation to be done in a sterile manner The nurses stated that it was important for basic hygiene to be upheld by all members of the OR staff “*The importance of sterility throughout the surgery and being responsible for ensuring that everyone in the OR follows the hygiene regulations*” “*Being well informed regarding the patient by reading their medical chart*” …it was important to have “*knowledge of postoperative wound infections in order to be able to prepare oneself properly*” Two of the nurses stated that they needed to be given the proper amount of time to prepare the skin disinfection of the surgical area, in order to allow them to perform their work well and without stress

### 2.6 Ethics

As this review synthesises previously published studies, no new ethical approval was required. The review was conducted in accordance with the principles of the Declaration of Helsinki and in accordance with copyright regulations.

## 3 Results

A total of 18 articles were included in the analysis and comprised studies from the following countries: Australia (*n* = 1), Canada (*n* = 1), Finland (*n* = 1), Iceland (*n* = 1), Iran (*n* = 1), Italy (*n* = 1), Norway (*n* = 2), Sweden (*n* = 8), the United Kingdom (UK) (*n* = 1) and an article with findings from the UK, Finland and the US (*n* = 1). Out of the total, 15 studies employed qualitative methods, while three used quantitative methods. The analysis yielded four categories: intrapersonal, interpersonal, adherence to guidelines and supportive conditions. These categories emerged from the subcategories: having control, planning ahead, competency, collaboration, mutual respect, management and communication systems.

### 3.1 Intrapersonal

The intrapersonal category included prerequisites among operating room nurses, focusing on beliefs, perceptions, emotions and personal characteristics related to prevention interventions during the intraoperative phase. The intrapersonal category was based on having control, planning ahead and competency.

#### 3.1.1 Having Control

The subcategory *Having control* was addressed in 14 of the 18 included studies and highlights how operating room nurses strive to maintain control over the environment, equipment and team behaviour to ensure patient safety and asepsis, while also describing various factors that challenged or supported their ability to retain such control.

To protect the patient and maintain an aseptic environment, the operating room nurses strove to have control over the operating room, the equipment and the personal actions of other professionals within their team (Wistrand et al. 2022; Lingard et al. 2004; Nyberg et al. 2022; Sandelin et al. 2019; Duff et al. 2022; Bastami et al. 2022; Aholaakko 2011; Qvistgaard et al. 2019). The operating room nurses were particularly observant of team members they did not know or trust (Aholaakko 2011). They actively worked to maintain control of the aseptic environment by implementing infection prevention interventions, including the replacement of non‐sterile equipment, glove changes, proper dressing application and ensuring the secure placement of drapes (Wistrand et al. 2018, 2022; Björn and Lindberg Boström 2008; Bastami et al. 2022). The aspiration was to have control over the equipment, ensuring its proper setup and coverage before the patient's arrival (Wistrand et al. 2022). There were factors that influenced operating room nurses' ability to have control. Feelings of an inability to cope with work demands were experienced (Sandelin et al. 2019; Alfredsdottir and Bjornsdottir 2008; Aholaakko 2011; Holmes et al. 2020). Lack of time was one major source of pressure, and operating room nurses often experienced time constraints when carrying out their clinical duties (Nyberg et al. 2022; Sandelin et al. 2019; Alfredsdottir and Bjornsdottir 2008; Aholaakko 2011; Kaldheim and Slettebø 2016). Pressure arose when they felt obligated to minimise the time allocated for mandatory tasks during preparation and surgery (Sandelin et al. 2019; Alfredsdottir and Bjornsdottir 2008; Aholaakko 2011; Holmes et al. 2020; Kaldheim and Slettebø 2016). Stress occurred when they felt the need to have ‘split vision,’ such as planning the next surgery while controlling sterility or performing infection prevention interventions (Silén‐Lipponen et al. 2005; Aholaakko 2011; Holmes et al. 2020). Worries could arise when team members neglected hygiene protocols or disregarded feedback regarding their behaviour (Nyberg et al. 2022; Silén‐Lipponen et al. 2005; Aholaakko 2011; Qvistgaard et al. 2019). Some operating room nurses explained how they took clear responsibility for maintaining aseptic control and resisted being influenced by team members or time pressure (Duff et al. 2022; Holmes et al. 2020). Factors related to the patient, such as inadequate preoperative preparations and physical challenges, were identified as infection risks and could cause concern, particularly among inexperienced operating room nurses (Aholaakko 2011).

#### 3.1.2 Planning Ahead

The subcategory *Planning ahead* was supported by the findings from nine of the 18 included studies, highlighting the importance of preparation as a prerequisite for ensuring safe and efficient infection prevention interventions. Various factors were described as influencing the ability to plan effectively.

The ability to plan ahead was essential for the operating room nurses (Wistrand et al. 2022; Björn and Lindberg Boström 2008; Nordström and Wihlborg 2019; Nyberg et al. 2022; Sandelin and Gustafsson 2015; Sandelin et al. 2019; Alfredsdottir and Bjornsdottir 2008; Kaldheim and Slettebø 2016). To be prepared meant being one step ahead (Björn and Lindberg Boström 2008). A precise preoperative plan made preparation easier and was found to reduce the patient's time in the operating room (Wistrand et al. 2022; Nyberg et al. 2022). A desire was expressed to review the patient's medical chart or preoperative plan before the procedure, ensuring that all necessary information and preparations were in place (Wistrand et al. 2022; Sandelin and Gustafsson 2015; Aholaakko 2011). Knowledge of patients' status and medical histories provided a higher level of awareness of risk factors such as vulnerability and fragility (Sandelin and Gustafsson 2015; Alfredsdottir and Bjornsdottir 2008). A preoperative conversation with the patient was highly advantageous, as the operating room nurses found it helped to ensure the provision of safe care (Sandelin and Gustafsson 2015). The operating room nurses felt that time constraints often hindered their ability to plan ahead as desired (Wistrand et al. 2022; Nordström and Wihlborg 2019; Nyberg et al. 2022; Sandelin et al. 2019; Aholaakko 2011).

#### 3.1.3 Competency

The subcategory *Competency* was supported by the findings from 10 of the 18 included studies, emphasising that operating room nurses' professional competence was a prerequisite for infection prevention interventions. Several factors were identified as influencing the development and application of professional competence in practice.

Operating room nurses' professional competence was considered a prerequisite for infection prevention interventions (Wistrand et al. 2022; Nyberg et al. 2022; Sandelin et al. 2019). Prevention was an essential part of their professional responsibility (Alfredsdottir and Bjornsdottir 2008) and infection prevention interventions were experienced as important to prevent surgical site infections (Wistrand et al. 2022; Qvistgaard et al. 2019). The operating room nurses' knowledge of surgical site infections was perceived as essential (Wistrand et al. 2022). They described their unique role within the surgical team, emphasising their responsibility for ensuring patient safety through the prevention of surgical site infections (Qvistgaard et al. 2019). The operating room nurses applied their knowledge to take charge of educating other professionals in surgical site infection prevention (Sandelin and Gustafsson 2015; Qvistgaard et al. 2019). Awareness of the sterile field, the environment and the consequences of actions was developed through experience (Duff et al. 2022). One study acknowledged the significance of both competence and experience (Qvistgaard et al. 2019). Having courage, being independent, along with competence, were considered prerequisites for directing actions in an aseptic environment—an ability that improved with experience (Aholaakko 2011; Qvistgaard et al. 2019). A friendly leadership style was perceived as effective (Sandelin and Gustafsson 2015; Holmes et al. 2020). The importance of being able to give constructive criticism (Kaldheim and Slettebø 2016) and not being confrontational was highlighted (Duff et al. 2022). Competence was perceived to emerge with 2–4 years of experience in the profession, and a distinction between competence and expertise was described (Aholaakko 2011). Inexperienced nurses could feel threatened by their lack of experience, and some expressed a desire to perform procedures as proficiently as more experienced colleagues (Aholaakko 2011).

### 3.2 Interpersonal

The Interpersonal category contains prerequisites for infection prevention interventions that were influenced by the interaction of two or more people. The interpersonal category was based on the subcategories of collaboration and mutual respect.

#### 3.2.1 Collaboration

This subcategory was supported by findings from 14 of the included studies and highlights that collaboration within the operating room team was a prerequisite for infection prevention interventions. Several factors influenced how collaboration was established and maintained in practice.

Collaboration was a prerequisite and involved other operating room nurses, surgeons, nurse assistants and anaesthetic nurses. The operating room nurses stated that all team members needed to understand the importance of infection prevention and adhere to hygiene rules (Wistrand et al. 2022; Duff et al. 2022; Qvistgaard et al. 2019). The importance of a well‐functioning team was emphasised (Nordström and Wihlborg 2019; Sandelin and Gustafsson 2015; Silén‐Lipponen et al. 2005; Aholaakko 2011; Qvistgaard et al. 2019; Holmes et al. 2020; Kaldheim and Slettebø 2016). Positive relationships among team members were found to facilitate infection prevention interventions and encouraged operating room nurses to speak up about errors and risks when necessary (Aholaakko 2011; Qvistgaard et al. 2019; Holmes et al. 2020). Opportunities for mutual support and collaboration across professional boundaries were created when operating room nurses trusted the competencies and responsibilities of other team members (Silén‐Lipponen et al. 2005; Qvistgaard et al. 2019; Holmes et al. 2020; Kaldheim and Slettebø 2016). The operating room nurses found it easier to work with people they knew (Kaldheim and Slettebø 2016). When two operating room nurses could collaborate, it was greatly appreciated (Nyberg et al. 2022; Sandelin et al. 2019). When performing preventive infection interventions, the operating room nurses were dependent on the nurse assistants' willingness to cooperate (Sandelin and Gustafsson 2015). It was highly appreciated when the surgeon was willing to help with infection prevention interventions (Holmes et al. 2020; Kaldheim and Slettebø 2016). The operating room nurses described the importance of reminding everyone to slow down, emphasising the need for new colleagues and students to be given extra time to perform their tasks (Nordström and Wihlborg 2019).

However, the operating room nurses described conditions when collaboration was ineffective. They suspected that other team members lacked knowledge of infection prevention (Duff et al. 2022). Necessary preoperative information was often lacking, and they sometimes had to double‐check information (Nyberg et al. 2022; Sandelin et al. 2019; Alfredsdottir and Bjornsdottir 2008; Holmes et al. 2020). Tensions in the team, unnecessary disturbances and poor communication were other conditions that negatively influenced their performance (Sandelin and Gustafsson 2015; Qvistgaard et al. 2019; Holmes et al. 2020; Kaldheim and Slettebø 2016). A lack of trust, communication, or understanding of each other's responsibilities could lead to risky events (Silén‐Lipponen et al. 2005; Qvistgaard et al. 2019; Holmes et al. 2020; Kaldheim and Slettebø 2016). When assessing the reasons for tensions in the operating room, safety and sterility were not prominent concerns (Lingard et al. 2004). Poor behaviour from team members negatively impacted their practice (Sandelin and Gustafsson 2015; Aholaakko 2011; Holmes et al. 2020; Kaldheim and Slettebø 2016). Irritable team members, particularly surgeons, could induce nervousness, which could lead to errors (Silén‐Lipponen et al. 2005; Qvistgaard et al. 2019; Holmes et al. 2020; Kaldheim and Slettebø 2016). For reasons of patient safety, the operating room nurses endeavoured to remain focused and not respond to any distractions or engage with any instances of bad behaviour (Silén‐Lipponen et al. 2005). Fearful of conflicts, they sometimes avoided informing the surgeon of poor aseptic practices (Lingard et al. 2004; Timmons and Tanner 2005; Aholaakko 2011). In one study, the operating room nurses said they needed to ensure that the surgeon had a smooth experience during surgery (Timmons and Tanner 2005).

#### 3.2.2 Mutual Respect

This subcategory is supported by findings from nine studies and emphasises mutual respect from the team as a prerequisite for operating room nurses' responsibility and implementation of infection prevention interventions. These prerequisites vary in practice and were at times challenged or lacking.

The operating room nurses wanted to be treated as equal team members who have valuable duties that take time (Björn and Lindberg Boström 2008; Nordström and Wihlborg 2019; Sandelin and Gustafsson 2015; Sandelin et al. 2019; Kaldheim and Slettebø 2016; Prati and Pietrantoni 2014). They felt that infection prevention interventions were perceived as having little value by other team members (Björn and Lindberg Boström 2008; Sandelin et al. 2019; Qvistgaard et al. 2019; Kaldheim and Slettebø 2016; Prati and Pietrantoni 2014). They described how they were not listened to when they pointed out shortcomings in adherence to hygiene rules (Duff et al. 2022). They described feeling drained after repeatedly highlighting the risk of infection and not being heard (Aholaakko 2011). Hierarchy was perceived to influence decisions regarding SSI prevention (Aholaakko 2011; Qvistgaard et al. 2019). The operating room nurses perceived that team members were annoyed when they called out inadequate aseptic practices or initiated aseptic measures. Nevertheless, their willingness to point out deficiencies could also be interpreted as demonstrating their competence in their profession (Nyberg et al. 2022).

### 3.3 Adherence to Guidelines

This subcategory is supported by the findings from 11 included studies and encompasses the prerequisites for integrating current recommendations into clinical practice, highlighting conflicting findings. The operating room nurses highlighted the importance of keeping well‐informed about new research, procedures and changes (Nordström and Wihlborg 2019). They reported believing that most recommendations in infection prevention guidelines were implemented and followed in their daily work (Nyberg et al. 2022). However, they also described occasions where guidelines were not adhered to—either by themselves, other operating room nurses, or team members (Wistrand et al. 2022; Lingard et al. 2004; Nyberg et al. 2022; Silén‐Lipponen et al. 2005; Timmons and Tanner 2005; Bastami et al. 2022; Aholaakko 2011; Qvistgaard et al. 2019). Adherence to recommendations was influenced by the surgeon, whom the operating room nurses believed held decision‐making authority (Silén‐Lipponen et al. 2005; Timmons and Tanner 2005). The operating room nurses could accommodate the surgeon's specific requests, including deviations from guidelines and manufacturer recommendations (Silén‐Lipponen et al. 2005; Timmons and Tanner 2005).

The operating room nurses perceived that interventions were performed based on their own experience, ward routines, or information from the medical chart (Nyberg et al. 2022; Sandelin and Gustafsson 2015). Knowledge of existing recommendations varied among the operating room nurses in a questionnaire study, with most being aware of guidelines for preoperative hand disinfection, while few knew whether guidelines existed for double‐glove use. The operating room nurses reported learning skin disinfection primarily from supervisors during clinical practice and specialist education (Wistrand et al. 2018).

### 3.4 Supportive Conditions

The supportive conditions category included prerequisites beyond the operating room nurses and their team, encompassing both the physical context and leadership. The category was based on management and communication systems.

#### 3.4.1 Management

This subcategory is supported by the findings from 11 studies, highlighting the importance of management and environmental factors as crucial prerequisites for infection prevention interventions in the operating room. The operating room nurses recognised good management as a crucial support for infection prevention interventions (Qvistgaard et al. 2019; Holmes et al. 2020). They believed that management should allocate sufficient time for preparation and infection prevention interventions (Wistrand et al. 2022; Nordström and Wihlborg 2019; Alfredsdottir and Bjornsdottir 2008). The operating room department's attitude towards hygiene standards was perceived to influence how the work was carried out (Duff et al. 2022). The operating room nurses did not always feel supported by management (Duff et al. 2022) and expressed a desire for management to better understand the situations they faced and to communicate their resource needs to the organisation (Duff et al. 2022; Kaldheim and Slettebø 2016). Poor management could lead to unstable routines and impede preventive interventions (Sandelin et al. 2019; Holmes et al. 2020).

The environment was described as a prerequisite for infection prevention interventions (Duff et al. 2022). Usually, the operating room nurses felt that there were good prerequisites for an aseptic environment (Nyberg et al. 2022; Prati and Pietrantoni 2014). The operating room nurses required a calm environment with minimal personnel and no door openings during both the preparation phase and the surgical procedure (Wistrand et al. 2022). The operating room nurses stressed the need for all members of the team to have access to appropriate personal equipment such as surgical suits, gloves, helmets and masks (Wistrand et al. 2022). Changes to timetables and team composition posed challenges in preparation (Sandelin et al. 2019; Alfredsdottir and Bjornsdottir 2008). Staffing imbalances, whether due to too few or too many team members, could lead to disorganised preparation or distraction (Aholaakko 2011).

#### 3.4.2 Communication Systems

This subcategory is supported by the findings from five of the included studies and highlights the essential role of well‐functioning communication systems as prerequisites for infection prevention interventions. Existing systems could show notable shortcomings.

Well‐functioning communication systems were described as prerequisites for effective infection prevention intervention (Wistrand et al. 2022; Nyberg et al. 2022; Alfredsdottir and Bjornsdottir 2008; Qvistgaard et al. 2019). The operating room nurses explained that sufficient preoperative information would ensure patient‐centred nursing, continuity of care and more efficient preparation (Alfredsdottir and Bjornsdottir 2008). However, they reported receiving insufficient and overly brief information from the anaesthesia assessment or the operating room planning system (Nyberg et al. 2022; Sandelin et al. 2019; Alfredsdottir and Bjornsdottir 2008). Also, a lack of an effective communication and distribution system was reported, with information about new practices or complications not reaching the appropriate personnel (Nyberg et al. 2022). Feedback systems on surgical site infection rates or complications were requested to assess the effectiveness of infection prevention interventions and to gain insights into the efficiency of routines (Wistrand et al. 2022; Nyberg et al. 2022). The operating room nurses emphasised that the absence of feedback systems for surgical site infections, as their efforts did not produce measurable results (Nyberg et al. 2022; Qvistgaard et al. 2019).

## 4 Discussion

This integrative review identified four categories of prerequisites that impact operating room nurses' ability to perform safe infection prevention interventions. These categories included intrapersonal prerequisites of individual operating room nurses, interpersonal prerequisites within the team, adherence to guidelines and supportive conditions.

Preventing surgical site infection was a high priority, and the operating room nurses guarded the environment to protect the patient. This control is comparable to the attributes of patient advocacy, where healthcare professionals make sure that patients receive the best possible care and that their rights and interests are respected and protected (Abbasinia et al. 2020). The operating room nurse plays an essential role in advocating for the patient's safety during surgical procedures. This striving for control is also similar to the concept of nursing vigilance, where nurses are meant to be scientifically, intellectually and experientially aware of the situation, to assess the risks and be prepared to minimise and respond to those risks (Meyer and Lavin 2005). As identified in this review, professional competence was crucial, not only for practising nursing vigilance but also for successfully implementing infection prevention interventions. The operating room nurses emphasised their unique role and knowledge regarding aseptic and infection prevention interventions within the team. This theoretical and clinical competence has been described as essential for ensuring patient safety during surgery (Von Vogelsang et al. 2019). Given this unique competence, operating room nurses are ideally positioned to lead infection prevention efforts within the team for patient safety and, therefore, must be given the necessary respect, time and resources for this duty. As previous studies have found, the competence of operating room is linked to knowledge, good communication skills and teamwork (Gillespie, Chaboyer, Wallis, Chang, and Werder 2009). This review emphasises the importance of teamwork as a prerequisite for infection prevention, which is consistent with previous research on infection prevention in general (Association of Perioperative Registred Nurses 2017; World Health Organization 2018). It is crucial for all team members to follow infection prevention guidelines and take appropriate actions. A synergistic effect can be achieved in the ideal team, where each individual contributes specific knowledge and competence to enhancing overall performance. Trust in the skills of other team members and good communication are prerequisites for infection prevention interventions. Previous studies have shown that inadequate information sharing during the intraoperative phase increases the risk of complications or patient mortality (Mazzocco et al. 2009). It has been suggested that operating room teams with consistent membership improve the quality of care, reduce conflicts and contribute to a flatter team hierarchy (Lingard et al. 2005; Makary et al. 2006). The extensive utilisation of temporary staff across several European nations raises concerns regarding patient safety, as it may have implications for effective communication, coordination and adherence to hygiene rules and routines (De Cuyper et al. 2019). Thus, from the perspective of patient safety, it is desirable to have more permanent teams.

This review revealed that operating room nurses perceived a lack of respect for their area of responsibility, including team members who did not follow hygiene rules and negative attitudes from surgeons with bad tempers. Disruptive behaviour in the operating room has been reported in previous studies (Rosenstein and O'Daniel 2008; Michael and Jenkins 2001). A study revealed that at least once per month, over 20% of operating room nurses reported experiencing recurring incidents of verbal abuse from physicians (Saridi et al. 2021). To manage this and to maintain a working relationship with specific surgeons, they have developed coping mechanisms, such as remaining silent after experiencing verbal abuse (Gillespie and Kermode 2004; Cochran and Elder 2015). Surgeons' disruptive behaviour has been shown to have a negative impact on patient safety by causing a loss of focus, increasing the risk of errors, reducing productivity and potentially leading to staff turnover (Cochran and Elder 2015; Lögde et al. 2018). These findings should serve as a wake‐up call for managers. Operating room nurses should feel empowered to report such behaviour and be confident that action will be taken to address it.

Furthermore, our findings addressed a hierarchical issue where the surgeon has ultimate decision‐making power over the responsibilities of operating room nurses. Similar dilemmas were described for midwives, who lacked the authority to make crucial decisions relating to births and faced ethical dilemmas because of hierarchical pressure from physicians and management (Türken and Çalım 2022). Even if surgeons have the formal responsibility for the surgery, they must follow established regulations and guidelines to ensure the safety and effectiveness of the procedure. For reasons of patient safety, operating room nurses must stop playing the role of ‘hostess’, as was described in this review and not act solely as a surgeon's assistant (Blomberg et al. 2015). Operating room nurses must stop fulfilling requests from surgeons that do not comply with evidence and guidelines. Another skill required might be the ability to challenge authority. For patient safety, operating room nurses must meet the expectations within their area of responsibility and thoroughly follow established evidence‐based practices. In prior studies, operating room nurses indicated that insufficient scientific evidence resulted in variations in skin preparation (Markström et al. 2020). Tradition‐based skin preparation has been observed during the intraoperative phase (Markström et al. 2020; Markström and Bjerså 2015). Developing more evidence on infection prevention interventions can support operating room nurses in their role as leaders of infection prevention interventions and may contribute to patient safety.

This review highlights the need for supportive conditions to ensure safe infection prevention interventions. Time constraints and a lack of information and feedback from management were reported. An earlier study examined the reasons why operating room nurses decided to remain in their workplace. It identified positive prerequisites, such as supportive leadership, personal stability and opportunities for personal and organisational growth, which are consistent with the findings of this review (Arakelian et al. 2019). The operating room nurses in this review often experienced stress and productivity demands. The concept of nurse resilience has gained increasing attention for its potential to inform strategies that support and enhance nurses' well‐being. Nurse resilience involves the ability of nurses to positively adapt to stress and adversity, incorporating both external resources and personal characteristics, which can vary with context and life circumstances (Cooper et al. 2020). The essential attributes that contribute to nursing resilience include social support from colleagues, managers, friends and families, as well as self‐belief, work‐life balance, self‐care, humour, optimism, realistic assessment of challenges and the establishment of attainable goals (Cooper et al. 2020). This review revealed that novice operating room nurses encountered higher levels of stress and uncertainty when carrying out infection prevention interventions. With the right support, younger and less experienced individuals can also experience resilience and growth in their profession, as one study highlighted that factors such as age, experience and education have a limited impact on the resilience of operating room nurses (Gillespie, Chaboyer, and Wallis 2009). In order to maintain their resilience, operating room nurses need to be able to identify and utilise their personal resources, receive support from management and be provided with working conditions that promote resilience. If management is not attentive to these needs, it may lead to a shortage of nurses and reduced patient safety.

## 5 Strengths and Limitations

This integrative review follows the methodological recommendations of Whittemore and Knafl (2005) and the preferred reporting items for systematic reviews (Page et al. 2021). Studies were reviewed and assessed using critical appraisal tools. Additionally, all five authors of this review jointly analysed and discussed the results in order to minimise subjective interpretation (Polit and Beck 2021).

Several methodological limitations are worth considering in this integrative review. The review includes studies from countries in Europe, as well as Australia, Canada and Iran, and the responsibilities of operating room nurses may differ between these. Thus, all the included studies provided insights into the operating room nurses' responsibility for aseptic practices and infection prevention interventions. Only 10 of the 18 included studies (55%) came from the database searches. This reflects a known issue related to indexing issues and inconsistent search terminology, especially in qualitative studies, which often require screening a large number of studies and the use of multiple search strategies (Whittemore and Knafl 2005; Shaw et al. 2004). A comprehensive literature search should include at least two or three search strategies (Conn et al. 2003). This review included five search strategies. Publication bias risk was present due to the selection of only English‐published studies, potentially leading to missed results. Initially, narrower search approaches were attempted, but these did not yield studies that aligned with the objectives of the study. In discussions with librarians, the broader data search strategy was chosen. However, it is important for the accuracy of the results of the integrative review that the literature research process is clearly and transparently documented and comprehensible (Whittemore and Knafl 2005). The inclusion of studies with lower methodological quality may be regarded as a limitation. Notably, these studies featured illustrative quotations from operating room nurses that offered insights directly relevant to the research question. It was assessed that the identified methodological shortcomings did not significantly compromise the relevance or credibility of the findings, as the limitations were not deemed to affect any aspects of the data utilised in the review.

## 6 Conclusions

In conclusion, from the perspective of operating room nurses, prerequisites for performing infection prevention interventions during the intraoperative phase included the professional competence of the operating room nurses, a supportive and understanding team and sufficient resources provided by management, including adequate equipment and time for planning and implementation. Furthermore, ensuring patient safety requires operating room nurses to embrace their role as leaders in infection prevention by adhering to evidence‐based recommendations.

## Author Contributions

The study's authorship was a collaborative effort involving all contributors. All authors actively participated in planning, goal setting, study design and completion of this integrative review. Following the initial planning phase, collaborative discussions among all authors were held to screen studies identified through data searches. Initial analysis was conducted by three authors, followed by discussions involving the remaining authors. The first author took the lead in manuscript writing, with each author providing feedback and contributing significantly. The final manuscript version underwent comprehensive review and approval by all authors.

## Funding

This study was funded by the County Council of Region Östergötland, Sweden.

## Ethics Statement

The authors have nothing to report.

## Consent

The authors have nothing to report.

## Conflicts of Interest

The authors declare no conflicts of interest.

## Supporting information


**Table S1:** An overview of the database searches conducted for this review, including search strategies and search.

**Table S2:** The quality appraisal of the included studies, presenting the assessment criteria and ratings used.

## Data Availability

The data that support the findings of this study are available from the corresponding author upon reasonable request.
