# Supplementary material for: Prerequisites for Infection Prevention Interventions During the Intraoperative Phase From the Perspective of Operating Room Nurses—An Integrative Review
Source: Nurs Open. 2026 Mar 19;13(3):e70498. doi: 10.1002/nop2.70498 (PMC13098050; doi:10.1002/nop2.70498)
Supplement: Supplementary file 1 — Table S1: An overview of the database searches conducted for this review, including search strategies and search. [file NOP2-13-e70498-s002.docx]

| Database | Main search | Limitations |
| --- | --- | --- |
| Pubmed  2022-04-12 | (patient safety OR patient harm) AND (perioperative nurse OR perioperative nursing OR operating room nurse OR operating room nursing OR operating theatre nursing OR operating theatre nurse)^1^ | English |
| Pubmed  2024-11-21 | (patient safety OR patient harm) AND (perioperative nurse OR perioperative nursing OR operating room nurse OR operating room nursing OR operating theatre nursing OR operating theatre nurse) AND ("2022/04/13"[Date - Create]: "3000"[Date - Create])^1^ | English |
| Cinahl  2022-04-12 | (patient safety OR patient harm) AND (perioperative nurse OR perioperative nursing OR operating room nurse OR operating room nursing OR operating theatre nursing)^2^ | English |
| Cinahl  2024-11-21 | ( (patient safety OR patient harm) AND (perioperative nurse OR perioperative nursing OR operating room nurse OR operating room nursing OR operating theatre nursing) ) AND EM 20220413^2^ | English |
| Embase  2022-04-12 | (patient safety OR patient harm) AND (perioperative nurse OR perioperative nursing OR operating room nurse OR operating room nursing OR operating theatre nursing)^2^ | English |
| Embase  2024-11-21 | ('patient safety'/exp OR 'patient safety' OR 'patient harm'/exp OR 'patient harm') AND ('perioperative nurse' OR 'perioperative nursing'/exp OR 'perioperative nursing' OR 'operating room nurse'/exp OR 'operating room nurse' OR 'operating room nursing'/exp OR 'operating room nursing' OR 'operating theatre nursing'/exp OR 'operating theatre nursing') AND [13-04-2022]/sd^2^ | English |
| Web of Science Core collection  2022-04-12 | ((patient safety OR patient harm) AND (perioperative nurse OR perioperative nursing OR operating room nurse OR operating room nursing OR operating theatre nursing)) ^3^ | English |
| Web of Science Core collection  2024-11-21 | ((patient safety OR patient harm) AND (perioperative nurse OR perioperative nursing OR operating room nurse OR operating room nursing OR operating theatre nursing)) AND LD=2022-04-13/2024-11-21^3^ | English |

**Table S1.** Overview of the database searches

(^1^ All fields, ^2^Default, ^3^Topic search)
