# Supplementary material for: Prerequisites for Infection Prevention Interventions During the Intraoperative Phase From the Perspective of Operating Room Nurses—An Integrative Review
Source: Nurs Open. 2026 Mar 19;13(3):e70498. doi: 10.1002/nop2.70498 (PMC13098050; doi:10.1002/nop2.70498)
Supplement: Supplementary file 2 — Table S2: The quality appraisal of the included studies, presenting the assessment criteria and ratings used. [file NOP2-13-e70498-s001.docx]

**Table** **S2. Quality appraisal of included qualitative and quantitative studies**

| Qualitative | | | | | | | | | | | |
| --- | --- | --- | --- | --- | --- | --- | --- | --- | --- | --- | --- |
| Author (year) | 1.Was there a clear statement of the aims of the research? | 2.Is a qualitative methodology appropriate? | 3.Was the research design appropriate to address the aims of the research? | 4.Was the recruitment strategy appropriate to the aims of the research? | 5.Was the data collected in a way that addressed the research issue? | 6.Has the relationship between researcher and participants been adequately considered? | 7.Have ethical issues been taken into consideration? | 8.Was the data analysis sufficiently rigorous? | 9. Is there a clear statement of findings? | 10.How valuable is the research | Value (max: 20) |
| Aholaakko  (2011) | 2 | 2 | 2 | 2 | 2 | 0 | 2 | 0 | 2 | 2 | 16 |
| Alfredsdottir & Bjornsdottir (2008) | 2 | 2 | 2 | 2 | 2 | 2 | 2 | 2 | 2 | 2 | 20 |
| Bastami et al.  (2022) | 2 | 2 | 2 | 2 | 1 | 0 | 1 | 0 | 1 | 1 | 14 |
| (Björn &Lindberg Boström (2008) | 2 | 2 | 2 | 2 | 2 | 2 | 2 | 2 | 2 | 2 | 20 |
| Duff et al  (2022) | 2 | 2 | 2 | 2 | 2 | 2 | 2 | 2 | 2 | 1 | 19 |
| Holmes, et al.  (2020) | 2 | 2 | 2 | 0 | 2 | 0 | 2 | 2 | 2 | 2 | 16 |
| Kaldheim & Slettebø  (2016) | 0 | 2 | 2 | 2 | 0 | 0 | 2 | 2 | 2 | 2 | 16 |
| Lingard, Garwood et al. (2004) | 2 | 2 | 2 | 2 | 2 | 2 | 2 | 2 | 2 | 2 | 20 |
| Nordström & Wihlborg (2019) | 2 | 2 | 2 | 2 | 2 | 0 | 2 | 2 | 2 | 2 | 18 |
| Nyberg, Olofsson et al. (2021) | 2 | 2 | 2 | 2 | 2 | 2 | 2 | 0 | 2 | 2 | 18 |
| Qvistgaard, Lovebo et al. (2019) | 2 | 2 | 2 | 2 | 2 | 0 | 2 | 0 | 2 | 2 | 16 |
| Sandelin and Gustafsson (2015) | 2 | 2 | 2 | 2 | 2 | 0 | 2 | 2 | 2 | 2 | 18 |
| Sandelin et al.  (2019) | 2 | 2 | 2 | 2 | 2 | 0 | 2 | 2 | 2 | 2 | 18 |
| Silén-Lipponen et al.  (2005) | 2 | 2 | 2 | 1 | 2 | 2 | 2 | 2 | 2 | 2 | 19 |
| (Timmons and Tanner (2005) | 1 | 2 | 1 | 2 | 1 | 0 | 2 | 1 | 2 | 2 | 14 |

*Adapted from the CASP checklist guidelines, gradings used; Yes, Can´t tell /not applicable or No. Numerical values ​​to each answer Yes = 2, Can´t tell/ not applicable = 1, No = 0.

For the qualitative studies, the maximum quality score was 20, 15-16 was moderate, and below 15 was considered low quality.

| Quantitative | | | | | | | | | | | | |
| --- | --- | --- | --- | --- | --- | --- | --- | --- | --- | --- | --- | --- |
| Authors (year) | 1.Did the study address a clearly focused issue? | 2. Did the authors use an appropriate method to answer their question? | 3. Were the subjects recruited in an acceptable way? | 4. Were the measures accurately measured to reduce bias? | 5.Were the data collected in a way that addressed the research issue? | 6. Did the study have enough participants to minimise the play of chance? | 7. How are the results presented and what is the main results? | 8.Was the data analysis sufficiently rigorous? | 9.Is there a clear statement of findings?. | 10. Can the results be applied to the local population? | 11. How valuable is the research? | Value (max: 22) |
| Prati & Pietrantoni (2014) | 2 | 2 | 1 | 2 | 2 | 2 | 2 | 2 | 2 | 0 | 1 | 18 |
| Wistrand et al. (2018) | 2 | 2 | 2 | 2 | 2 | 1 | 2 | 2 | 2 | 2 | 2 | 21 |
| (Wistrand et al. (2021) | 2 | 2 | 2 | 2 | 2 | 1 | 2 | 2 | 2 | 2 | 2 | 21 |

*Adapted from the CASP checklist guidelines, gradings used: Yes, Can´t tell /not applicable or No. Numerical values ​​to each answer Yes = 2, Can´t tell/ not applicable = 1, No = 0

For the quantitative studies, maximum quality score was 22. A score between 17-18 was considered moderate, and below 17 was considered low quality.
